# Physical activity and not sedentary time per se influences on clustered metabolic risk in elderly community-dwelling women

**DOI:** 10.1371/journal.pone.0175496

**Published:** 2017-04-07

**Authors:** Andreas Nilsson, Britta Wåhlin-Larsson, Fawzi Kadi

**Affiliations:** School of Health Sciences, Örebro University, Örebro, Sweden; University of Bath, UNITED KINGDOM

## Abstract

**Introduction:**

Whether amount of time spent in sedentary activities influences on clustered metabolic risk in elderly, and to what extent such an influence is independent of physical activity behavior, remain unclear. Therefore, the aim of the study was to examine cross-sectional associations of objectively assessed physical activity and sedentary behavior on metabolic risk outcomes in a sample of elderly community-dwelling women.

**Methods:**

Metabolic risk outcomes including waist circumference, systolic and diastolic blood pressures, fasting levels of plasma glucose, HDL-cholesterol and triglycerides were assessed in 120 community-dwelling older women (65–70 yrs). Accelerometers were used to retrieve daily sedentary time, breaks in sedentary time, daily time in light (LPA) and moderate-to-vigorous physical activity (MVPA), and total amount of accelerometer counts. Multivariate regression models were used to examine influence of physical activity and sedentary behavior on metabolic risk outcomes including a clustered metabolic risk score.

**Results:**

When based on isotemporal substitution modeling, replacement of a 10-min time block of MVPA with a corresponding time block of either LPA or sedentary activities was associated with an increase in clustered metabolic risk score (β = 0.06 to 0.08, p < 0.05), and an increase in waist circumference (β = 1.78 to 2.19 p < 0.01). All associations indicated between sedentary time and metabolic risk outcomes were lost once variation in total accelerometer counts was adjusted for.

**Conclusions:**

Detrimental influence of a sedentary lifestyle on metabolic health is likely explained by variations in amounts of physical activity rather than amount of sedentary time per se. Given our findings, increased amounts of physical activity with an emphasis on increased time in MVPA should be recommended in order to promote a favorable metabolic health profile in older women.

## Introduction

While beneficial effects of physical activity (PA) for prevention and treatment of cardio-metabolic disorders are well-documented [1], a growing body of research focuses on the proposed detrimental role of sedentary behavior on different health-related outcomes. Sedentary behavior is defined as any waking behavior with an energy expenditure ≤ 1.5 metabolic equivalents (METs) and a sitting or reclining posture [2]. Amount of time spent sedentary has been found associated with both increased all-cause mortality and cardiovascular disease (CVD) mortality, as well as increased risk of diabetes and presence of the metabolic syndrome [3, 4, 5], the latter defined as a cluster of metabolic disorders including abdominal obesity, hypertension, dyslipidemia and hyperglycemia. Besides total amount of sedentary time, the frequency of breaks in sedentary time has been reported associated to level of adiposity, HDL-cholesterol, and likelihood of having the metabolic syndrome [6, 7, 8, 9], where a low break rate leading to more prolonged time periods of sedentary behavior seems related to detrimental effects on health outcomes.

A number of studies have reported associations between sedentary time and metabolic risk outcomes independently of time spent in MVPA [10], recognizing sedentary time as a unique health risk, regardless of beneficial health effects induced by time spent in PA. However, this hypothesis has recently been questioned by others, where variation in total amount of PA was reported to attenuate the influence of sedentary time on mortality risk [11], and metabolic risk outcomes [12], indicating sedentary time simply to reflect lower volumes of PA. To date, surprisingly few studies have reported on the potentially confounding effects of objectively assessed PA volume other than time in MVPA on the proposed influence of sedentary behavior on metabolic risk outcomes. Furthermore, in a number of studies adjustments for measures of adiposity (e.g. body mass index or waist circumference) attenuated associations between sedentary time or breaks in sedentary time and components related to the metabolic syndrome (e.g. blood lipid levels) [10], which further questions the role of sedentary time as an independent health risk.

Notably, a recent review on previous work based solely on elderly populations summarized the influence of sedentary behavior on metabolic risk as currently inconclusive [13), where differences between studies regarding assessment methods (objective or self-report), definition of sedentary behavior (e.g. accumulated or continuous bouts, breaks in sedentary time), heterogeneity of participants’ health status, and handling and analysis of data at least partly can explain these inconclusive findings [13]. Furthermore, relatively few studies addressing influences of PA and sedentary behavior on metabolic risk outcomes are based on older populations specifically. The paucity of studies on older groups is unfortunate as the prevalence of the metabolic syndrome and concomitant risk of CVD raises substantially with increasing age, where a 5-fold increased prevalence of the metabolic syndrome occurred in groups of women in a large-scale population from ages 19–39 years to 60–78 years [14]. Further, older individuals spend more time in sedentary behaviors and less time in health-enhancing PA compared to younger age groups [15], and a larger portion of women than men do not meet current guidelines on minimum levels of weekly PA [16], making the elderly population in general and elderly women in particular a target of public health interest.

Taken together, whether time in sedentary activities influences on metabolic risk in elderly, and if so to what extent such an influence is independent of PA behavior, remain currently unclear. Elderly community-dwelling women generally show low PA levels and at the same time high prevalence rates of the metabolic syndrome. Given this, increased knowledge of how PA and sedentary behavior relates to metabolic health among elderly community-dwelling women is warranted in order to develop strategies for successful promotion of healthy aging for this least physically active group of the population.

Therefore, the aim of the present study was to examine associations between objectively assessed PA and sedentary behaviors with metabolic risk outcomes in a sample of elderly community-dwelling women.

## Materials and methods

### Participants

A total of 120 elderly community-dwelling women, 65–70 yrs, were recruited through an advertisement in a local newspaper. The narrow age range was selected as part of inclusion criteria to a randomized controlled trial previously reported elsewhere [17]. All women were living in an urban area in Sweden and of white European origin. All women included in the study were free of diagnosed coronary heart disease or diabetes mellitus, had no disability in regard to mobility, and were non-smokers. Written informed consent was obtained from all participants. All clinical investigation was conducted according to the principles expressed in the Declaration of Helsinki. The study was approved by ethical committee, the regional ethical review board, in Uppsala, Sweden (2011/033).

### Assessment of anthropometrics and metabolic risk outcomes

Height measured to the nearest 0.5 cm and body weight measured to the nearest 0.1 kg were assessed by a portable stadiometer and a digital scale, respectively. Metabolic risk outcomes variables included in the IDF definition of the metabolic syndrome [18] were assessed: Waist circumference (WC) was measured to the nearest 0.1 cm with a steel tape at the midpoint between iliac crest and lower costal margin. Systolic and diastolic blood pressures were measured manually after a 15-minute rest in the supine position using a mercury sphygmomanometer. A blood sample was collected after an overnight fast by venipuncture from an antecubital vein. Levels of triglycerides and HDL-cholesterol were determined on a Vitros-5.1 analyser platform using chemistry kits from Ortho-Clinical Diagnostics, Johnson & Johnson. Level of plasma glucose was determined with the Roche Reflotron Plus^®^ system.

A continuous clustered metabolic risk score (zMS) was created based on the assessed metabolic risk outcome variables (level of triglycerides, HDL-cholesterol, plasma glucose, waist circumference and mean blood pressure). First, standardized values (z-scores) of each outcome variable were expressed, and thereafter the average z-score based on all standardized outcome variables were calculated. In order to examine whether the influence of times in PA and sedentary behavior on clustered metabolic risk are mainly driven by abdominal obesity, we additionally calculated a clustered metabolic risk score without the waist circumference component included (zMS-wc). Z-scores on HDL-cholesterol were inverted before summing with the other outcome variables.

### Assessment of physical activity and sedentary behavior

Data on PA and sedentary behavior was assessed by the Actigraph GT3x (Actigraph, Pensacola, Florida) activity monitor during a week. The monitor was worn tightly fastened with an elastic belt at the right hip (iliac crest) and 60s epochs were used. All participants were instructed to wear the monitor at all waking hours, with exception for water activities, and note all events when the monitor was removed during awake time, and note the time on and off for sleep times at night. At least 4 days with at least 10 hours of wear time per day was required for inclusion. Non-wear time was defined as a minimum of 60 min of continuous zero counts. Counts >20.000 counts per minute were discarded as non-human movements. Count cut points for sedentary time (<100 counts per min), time in LPA (100–2019 counts per min), and MVPA (>2019 counts per min) were applied in accordance with previous work [19, 20] to retrieve daily times spent in those activity categories. Two measures of daily sedentary time was retrieved; total accumulated time and the amount of time occurring in bouts of at least 10 consecutive minutes, where the latter has been showed to capture the prolonged nature of sedentary behavior and its relation to health outcomes when using accelerometers [21]. A one-minute epoch of ≥100 counts following sedentary time was regarded a break in sedentary time. Total daily number of breaks over total daily sedentary time was calculated and referred to as break rate. We also calculated total counts minus number of counts derived during sedentary activities (i.e. < 100 counts per min), which was used to investigate potential influence of variation in total level of PA on the relationships between sedentary time and metabolic risk outcomes.

### Assessment of covariates

Information on medical history, including current use of medical drugs, were collected by a physician during visit for blood sample assessment. Self-rated health status was assessed by questionnaire (SF-12). Data on energy intake was assessed by a six-day food record, where data on total energy intake, fat intake, and alcohol consumption were derived.

### Statistical analysis

Data are presented as means ± SD. All data variables were checked for normality and log transformed if necessary to fit a normal distribution.

Multivariate linear regression analyses were used to examine the influence of sedentary behaviors (total sedentary time, sedentary time in bouts of at least 10 consecutive minutes, and average break rate), time in LPA and MVPA on metabolic risk outcomes. All time based variables were expressed in 10-minute blocks to represent a time frame in line with PA guidelines. Influence of time in PA and sedentary activities on metabolic risk outcomes was first assessed in single models, and additionally adjusted for either sedentary time (when modeling time in LPA or MVPA) or time in MVPA and accelerometer counts derived during non-sedentary time periods (when modeling sedentary time). Adjustments for MVPA time or accelerometer counts were made separately as they were strongly correlated (r = 0.77; p < 0.01).

We further investigated the potential impact of displacing 10-minute periods in different time-based activity categories on metabolic risk outcomes based on isotemporal substitution modeling. In such a model, the regression coefficient represents the hypothetical change in metabolic risk outcome when reallocating time spent in one type of activity behavior with the corresponding time frame in another activity behavior, while holding total wear time constant.

Potential influence of intake of lipid lowering drugs and antihypertensive drugs, daily energy intake (kJ), fat intake (E%), alcohol consumption (g/day), and self-rated health (three categories) on metabolic risk outcomes were first checked using regression models with backwards elimination method, with p ≥ 0.1 set as F-to-remove criteria. Medication was dichotomized as ‘yes’/‘no’. All outcomes, except for clustered metabolic risk scores and WC, were finally adjusted for WC. Assumptions for regression models including linearity, homoscedasticity, and multicollinearity between independent variables were checked (VIF < 10). Notably, a VIF of 2.7 was the largest noted when modelling accumulated sedentary time together with accelerometer counts. All statistical analyses were performed using SPSS ver. 23. Level of statistical significance was set to p < 0.05, which allowed detection of smaller effect sizes (≤ 0.15) with a power of ≥ 80% when performing all regression models.

## Results

A total of 113 of the 120 recruited women (mean age 67.5 ± 1.6 yrs) had complete data on all outcome variables. Five women had incomplete accelerometer recordings and two women had incomplete data on metabolic risk outcomes. Thirty-five women (31%) used lipid-lowering or antihypertensive drugs. The average number of monitored days with the accelerometer was 5.8 ± 0.5 days, with a mean wear time of 14.2 ± 1.0 hours per day. Subject characteristics on all 113 women are shown in Table 1.

**Table 1:** Subject characteristics based on 113 women.

Variables	Mean ± SD
Height (cm)	164.7 ± 5.7
Body weight (kg)	69.2 ± 11.2
Waist circumference (cm)	84.4 ± 10.3
Systolic blood pressure (mmHg)	135.8 ± 14.9
Diastolic blood pressure (mmHg)	77.8 ± 8.5
Triglycerides (mmol/l)	1.13 ± 0.43
HDL-cholesterol (mmol/l)	1.58 ± 0.34
Glucose (mmol/l)	5.3 ± 0.6
Daily sedentary time (min)	518 ± 77
Daily sedentary time in bouts^a^ (min)	327 ± 79
Daily time in LPA (min)	294 ± 65
Daily time in MVPA (min)	37 ± 23
Break rate (breaks·SEDh^-1^)^b^	10.0 ± 2.0

^a^ Sedentary time in bouts of at least 10 consecutive minutes

^b^ Average number of breaks per sedentary hour

Influences of 10-minute blocks of time spent sedentary, derived either in an accumulated fashion or in continuous time bouts, and accumulated time in LPA and MVPA on metabolic risk outcomes are presented in Table 2. Variables on energy intake, fat intake, alcohol intake and self-rated health were not included in final models as criteria for removal (p ≥ 0.1) was met when modelling with backwards elimination method. Hence, final models were adjusted by age, monitor wear time, and intake of lipid lowering drugs and antihypertensive drugs (Table 2).

**Table 2:** Associations (β-coefficients, 95% CI) between accumulated sedentary time (accumulated SED-time), sedentary time in bouts of at least 10 consecutive minutes (continuous SED-time), time in LPA and MVPA, and metabolic risk outcomes (n = 113).

	Accumulated SED-time (min/day)	Continuous SED-time (min/day)	LPA (min/day)	MVPA (min/day)
*z*MS (*z*-score)^a^	0.02 (-0.00, 0.03)	0.02 (0.01 0.03)*	-0.01 (-0.02, 0.01)	-0.06 (-0.11, -0.02)*
*z*MS-wc (z-score)^b^	0.02 (-0.00, 0.03)	0.02 (0.01 0.03)*	-0.01 (-0.03, 0.01)	-0.03 (-0.08, -0.02)
Waist circumference (cm)	0.09 (-0.20, 0.39)	0.16 (-0.10 0.41)	0.16 (-0.14 0.45)	-1.83 (-2.56, 1.11)**
SBP (mmHg)	0.12 (-0.31, 0.54)	0.21 (-0.16 0.58)	-0.18 (-0.61, 0.25)	0.51 (-0.67, 1.68)
DBP (mmHg)	-0.06 (-0.30, 0.18)	0.02–0.19, 0.23	0.03 (-0.21 0.28)	0.21 (-0.45, 0.87)
Triglycerides (mmol/l)	0.00 (-0.02, 0.00)	0.01 (0.00, 0.02)*	-0.01 (-0.02, 0.00)	-0.01 (-0.04, 0.03)
HDL-cholesterol (mmol/l)	-0.00 (-0.02, 0.00)	-0.01 (-0.01, 0.00)	0.00 (-0.01, 0.01)	0.03 (0.01, 0.06)*
Glucose (mmol/l)	0.01 (-0.01, 0.03)	0.00 (-0.01, 0.02)	-0.01 (-0.02, 0.01)	-0.03–0.08, 0.01)

All coefficients are adjusted for age, monitor wear time, intake of lipid lowering and/or antihypertensive medication.

^a^ Clustered metabolic risk score

^b^Clustered metabolic risk score without waist circumference

**p < 0.01

*p < 0.05

Amount of sedentary time spent in bouts of at least 10 consecutive minutes was significantly associated to both scores representing clustered metabolic risk (zMS and zMS-wc) (Table 2). While these associations were independent of time in MVPA, with similar effect sizes in both models (β = 0.02 (95% CI: 0.01, 0.03) p < 0.05), none of these associations remained when adjusting for daily amount of accelerometer counts derived from non-sedentary activities (β = 0.01, p > 0.1 in both models). Triglyceride level was the only metabolic risk outcome to be significantly associated with continuous sedentary time (Table 2). While this association was independent of time in MVPA (β = 0.01 (95% CI: 0.00, 0.02) p < 0.05), it became non-significant when adjusting for daily amount of accelerometer counts (β = 0.01 (95% CI: -0.00, 0.02) p = 0.09). Moreover, adjustment for WC removed statistical significance in the relation between continuous sedentary time and triglyceride level (β = 0.01 (95% CI: -0.01, 0.02) p = 0.08), regardless of adjustment for PA.

No relations were observed between any of the metabolic risk outcomes and time in LPA. In contrast, time in MVPA was inversely associated with clustered metabolic risk (zMS) independent of sedentary time (β = -0.06 (95% CI: -0.11, -0.02) p < 0.05), whereas this association was lost when excluding WC from the clustered metabolic risk score (Table 2). As expected, a significant inverse association between MVPA and WC was observed independent of sedentary time (β = -1.86 (95% CI: -2.60, -1.11) p < 0.01). Furthermore, an influence of time in MVPA on level of HDL-cholesterol was indicated (Table 2), which remained independent of sedentary time (β = 0.03 (95% CI: 0.01, 0.06) p < 0.05). However, further adjustment for WC attenuated this association (β = 0.02, p > 0.1).

Isotemporal substitution analysis revealed a significant increase in clustered metabolic risk score (zMS) (p < 0.05) and WC (p < 0.01) when replacing a 10-min time block of MVPA with either LPA or time in sedentary activities, regardless of how the sedentary time was derived (Table 3). Importantly, when excluding the WC component from the risk score, a significant change in clustered metabolic risk was no longer evident when hypothetically replacing MVPA with accumulated sedentary time or time in LPA (Table 3). We additionally analyzed potential effects on both clustered metabolic risk scores when replacing a 10-minute block of sedentary time (either accumulated time or continuous time) with time in LPA, which revealed no related changes in either of the risk scores (p > 0.1 in both models).

**Table 3:** Associated change (β-coefficients, 95% CI) in clustered metabolic risk scores (*z*MS, *z*MS-wc) and waist circumference when replacing a 10-min time block of MVPA with LPA or time in sedentary activities (accumulated SED-time or continuous SED-time) (n = 113).

Replace MVPA time with:	*z*MS (z-score)	*z*MS-wc (z-score)	Waist circumference (cm)
LPA (min/day)	0.06 (0.01, 0.10)*	0.02 (-0.02, 0.07)	2.19 (1.45, 2.93)**
Accumulated SED-time (min/day)	0.07 (0.02, 0.11)**	0.04 (-0.01, 0.09)	1.78 (1.04, 2.57)**
Continuous SED-time (min/day)	0.08 (0.04, 0.13)**^a^	0.05 (0.01, 0.10)*^a^	2.08 (1.35, 2.8)**^a^

Adjusted for age, intake of lipid lowering and antihypertensive medication

^a^Sedentary time in bouts of less than 10 consecutive minutes have been accounted for in the model

**p < 0.01

*p < 0.05

Finally, break rate (breaks·SEDh^-1^) was inversely and significantly related to both scores on clustered metabolic risk independent of time in MVPA (zMS: β = -0.07 (95% CI: -0.12, -0.02) p < 0.05; zMS-wc: β = -0.07 (95% CI: -0.12, -0.01) p < 0.05). Both observed associations became attenuated after adjustment for daily amount of accelerometer counts (β = -0.03, p > 0.1 in both models). Further, an inverse association with triglyceride level was also observed independent of time in MVPA (β = -0.04 (95% CI: -0.07, -0.01) p < 0.05), which became attenuated after further adjustment for WC (β = -0.03 (95% CI: -0.06, 0.00) p = 0.08).

## Discussion

A salient finding in our study was that associations between sedentary behaviors and metabolic risk outcomes are not independent of PA. Hence, physiological mechanisms induced by variations in PA volume rather than variations in amount of sedentary time per se likely explain detrimental effects of a sedentary lifestyle on metabolic risk outcomes in elderly women. Furthermore, our data highlights the role of abdominal obesity as an important mediating factor between different metabolic risk outcomes and time spent in different activity categories in elderly women.

In contrast to the total amount of sedentary time accumulated during a day, our data indicated that the amount of sedentary time occurring in bouts of at least 10 consecutive minutes is associated with detrimental effects on metabolic health outcomes, which supports previous research [22, 23]. However, observed effect sizes of sedentary behavior on metabolic risk outcomes were small, and even though associations remained significant when controlling for MVPA time, none remained significant after controlling for total daily accelerometer counts. This is in line with data reported by Maher et al. [12] based on a large sample of adults from the U.S., where cross-sectional relations between total accumulated sedentary time and different cardio-metabolic risk factors were effectively attenuated when adjustments for variations in daily accelerometer counts were made. Similarly, in another cross-sectional study based on a Japanese population of middle-aged men and women, the increased risk of having the metabolic syndrome among those belonging to the highest tertile of sedentary time compared to the lowest tertile lost statistical significance once differences in time spent in PA of at least light intensity was controlled for [24].

Although no relation between time in PA below the MVPA threshold and metabolic risk was observed in the current study, a higher break rate was associated with a lower clustered metabolic risk, which also is in accordance with previous observations [9, 22, 23]. A high break rate mean less prolonged periods in sedentary activities and subsequently more opportunities to increase total volume of PA. A transition from a sedentary state to a more active behavior has in experimental settings been shown to induce changes in the expression of genes regulating muscle cell growth and proliferation, an increased fatty acid uptake into skeletal muscle with reduced plasma triglyceride levels, and a reduced postprandial glucose and insulin level [25, 26, 27], which all may impact on metabolic health status. Given this, the positive association between triglyceride level and continuous sedentary time indicated in the current study may be reflections of a favorable response of breaking up sedentary time periods. Thus, variations in PA volume rather than amount of sedentary time per se induce physiological mechanisms that may be detrimental to health in elderly.

As demonstrated by isotemporal substitution modelling, replacing a 10-minute time block of MVPA with any other activity of lesser intensity, was related to an increase in clustered metabolic risk score as well as a two centimeter average increase in WC in this sample of elderly women (Table 3). These results are in accordance with recent work reporting on changes in metabolic risk outcomes and mortality risk when replacing time in higher intensity PA with lower intensity activities [28, 29, 30]. For example, Hamer et al [28] reported similar changes on BMI and blood lipid levels when hypothetically replacing a 10-minute period of MVPA with either LPA or sedentary time in a sample of older men and women. Furthermore, Janssen et al [30] reported based on a large middle-aged population that individuals with the highest amount of time spent in vigorous intensity PA had a reduced risk of having the metabolic syndrome compared to those belonging to the group without any time spent in vigorous intensity PA, indicating that PA of higher intensity may be of importance in metabolic disease prevention. The chosen count cut-point for assessing MVPA time (i.e. 2020 cnts·min^-1^) in the current study makes it less likely that lower intensity activities, such as household chores, became included in the MVPA time category. Hence, our results indicate that MVPA corresponding to at least brisk walking may have stronger implications on metabolic risk than lower intensity PA also in elderly populations.

In our study, all observed associations between time spent in MVPA and single metabolic risk outcomes were attenuated by WC. Different body fat distributions have been shown to be associated with different metabolic risk levels. For example, Fox et al [31] reported on significant positive relations between blood lipid levels and amounts of adipose tissue, where the risk scores related to metabolic syndrome subcomponents were stronger for visceral fat depots than for subcutaneous fat depots [31]. While the underlying mechanisms explaining observed detrimental health effects remain to be solved, abdominal obesity with large visceral adipose tissue depots is suggested to be related to disturbed adipokine secretions, which in turn play an integral part in development of low-grade systemic inflammation and insulin resistance [32]. Although WC is a crude measure of both visceral and subcutaneous adipose depots around the waist, we still like to believe that WC rather than BMI will better reflect potential influence of adiposity in general, and visceral adipose tissue in particular, when modeling associations between PA behaviors and metabolic risk outcomes.

Similar to most previous work, we did not analyze times spent in PA intensities occurring in consecutive time bouts, even though current guidelines recommend episodes of MVPA to last for at least 10 minutes. However, it could be argued that examining accumulated time in MVPA regardless of bout lengths is more appropriate given the old age group in the current study, as maintaining longer episodes of MVPA may be more challenging in this age group compared to younger counterparts. Furthermore, a study based on a sample of older men recently showed that strength in associations between markers for metabolic risk and daily time in MVPA did not differ depending on whether time in MVPA was derived in consecutive time bouts or just accumulated over the day [33].

Finally, it is important to stress that the cross-sectional design of our study does not allow to infer causality. Moreover, being relatively small and homogenous with respect to age, ethnicity and demographic background, our sample is unlikely to be representative of broader populations of older women. Noteworthy, means and standard deviations on PA and sedentary behavior in this sample of elderly women are similar to what has been previously reported in studies based on larger populations including older women [15, 20]. Although the Actigraph accelerometer is a widely accepted tool for assessment of sedentary behaviors, with an ability to correctly identify more than 80% of sedentary activities in a sitting or lying position [34], sedentary behaviors with regard to posture cannot be readily assessed. Hence, lack of human movement rather than posture is assessed with highest accuracy in the present study. Also similar to most previous studies based on accelerometry, PA and sedentary behaviors were assessed during awake time rather than 24-hours, which means that some awake time periods, particularly if occurring during night times due to sleep disturbances, have been excluded. Although influences from unmeasured covariates cannot be excluded, we believe that exclusion of energy intake, fat intake, alcohol intake, and self-reported health status strengthens the observed associations as indicators of existing relationships between PA behavior and metabolic risk outcomes in elderly women. Increasing the sample size, and thus statistical power, would strengthen rather than attenuate these findings.

In conclusion, no associations between sedentary behavior and metabolic risk outcomes were evident once variation in accelerometer counts was controlled for. Therefore, amount of PA rather than amount of sedentary time per se influences on clustered metabolic risk in elderly women. Isotemporal substitution modeling showed that a hypothetical replacement of time in MVPA with activities of less intensity is related to an increased clustered metabolic risk, which is explained by an inverse relation between MVPA time and abdominal obesity. Given our findings, increased amounts of PA with an emphasis on increased time in MVPA should be recommended in order to promote a favorable metabolic health profile in elderly women.

## Supporting information

S1 Table Supplementary information on subject data. (DOCX)
