# Supplementary material for: Physical activity and not sedentary time per se influences on clustered metabolic risk in elderly community-dwelling women
Source: PLoS One. 2017 Apr 7;12(4):e0175496. doi: 10.1371/journal.pone.0175496 (PMC5384780; doi:10.1371/journal.pone.0175496)
Supplement: S1 Table — (DOCX) [file pone.0175496.s001.docx]

**Supplementary information**

S1 Table. Supplementary information on subject data

| Variables | Mean ± SD |
| --- | --- |
| Energy intake (kj) | 7125 ± 1537 |
| Fat intake (E%) | 34.2 ± 5.1 |
| Alcohol intake (g) | 7.5 ± 7.1 |
| Self-rated health (SF-12 index) | 50.9 ± 8.5 |
| - First tertile - Second tertile - Third tertile | 41.6 ± 7.1  53.1 ± 1.8  58.5 ± 3.0 |
